# Tyrosine, Cysteine, and *S-*Adenosyl Methionine Stimulate *In Vitro* [FeFe] Hydrogenase Activation

**DOI:** 10.1371/journal.pone.0007565

**Published:** 2009-10-26

**Authors:** Jon M. Kuchenreuther, James A. Stapleton, James R. Swartz

**Affiliations:** 1 Department of Chemical Engineering, Stanford University, Stanford, California, United States of America; 2 Department of Bioengineering, Stanford University, Stanford, California, United States of America

## Abstract

**Background:**

[FeFe] hydrogenases are metalloenzymes involved in the anaerobic metabolism of H_2_. These proteins are distinguished by an active site cofactor known as the H-cluster. This unique [6Fe–6S] complex contains multiple non-protein moieties and requires several maturation enzymes for its assembly. The pathways and biochemical precursors for H-cluster biosynthesis have yet to be elucidated.

**Principal Findings:**

We report an *in vitro* maturation system in which, for the first time, chemical additives enhance [FeFe] hydrogenase activation, thus signifying *in situ* H-cluster biosynthesis. The maturation system is comprised of purified hydrogenase apoprotein; a dialyzed *Escherichia coli* cell lysate containing heterologous HydE, HydF, and HydG maturases; and exogenous small molecules. Following anaerobic incubation of the *Chlamydomonas reinhardtii* HydA1 apohydrogenase with *S*-adenosyl methionine (SAM), cysteine, tyrosine, iron, sulfide, and the non-purified maturases, hydrogenase activity increased 5-fold relative to incubations without the exogenous substrates. No conditions were identified in which addition of guanosine triphosphate (GTP) improved hydrogenase maturation.

**Significance:**

The *in vitro* system allows for direct investigation of [FeFe] hydrogenase activation. This work also provides a foundation for studying the biosynthetic mechanisms of H-cluster biosynthesis using solely purified enzymes and chemical additives.

## Introduction

Hydrogenases are subdivided into three classes: [NiFe] hydrogenases, [FeFe] hydrogenases, and [Fe] hydrogenases, each characterized by a unique active site cofactor [1]–[5]. [NiFe] and [FeFe] hydrogenases catalyze the reversible oxidation of dihydrogen: H_2_⇆2H^+^+2e^−^. Of these, [FeFe] hydrogenases have intrinsically higher *in vitro* H_2_ evolution rates [6], making them more attractive candidates for production of H_2_ as a sustainable biofuel. The [FeFe] hydrogenase active site cofactor, known as the H-cluster, is composed of a conventional [4Fe–4S] cubane cluster joined by a cysteinyl sulfur to a unique [2Fe] sub-cluster that includes multiple non-protein ligands covalently attached to the sub-cluster iron atoms [6]. These non-protein moieties have been identified as carbon monoxide (CO), cyanide (CN) [2], and a putative dithiopropane or dithiomethylamine bridge [3], [7].

Three proteins required for active [FeFe] hydrogenase production – HydE, HydF (fused as HydEF in eukaryotes), and HydG – were first identified by analyzing *C. reinhardtii* mutants incapable of H_2_ photoproduction. Subsequent recombinant co-expression of the *C. reinhardtii* [FeFe] hydrogenase with *C. reinhardtii* HydEF and HydG in *E. coli* enabled production of active hydrogenase [8]. Following this discovery, *in vitro* work with the individual maturases has shed light on their respective roles in the synthesis of the H-cluster cofactor and its insertion into the hydrogenase active site. HydE and HydG, which both contain [Fe–S] clusters and sequence motifs generally attributed to radical SAM enzymes [8], have been shown to reductively cleave SAM to form 5′-deoxyadenosine [9]. Recently, SAM-dependent HydG activity was shown to increase in the presence of tyrosine, leading to a hypothesis that a tyrosine-derived dehydroglycine intermediate is the source for the H-cluster dithiol bridge [10]. HydF has been identified as a GTPase based on sequence alignment analysis [8], [11] and its ability to hydrolyze GTP to GDP [11]. In earlier efforts to reproduce apohydrogenase maturation, HydF was isolated after recombinant co-expression with HydE and HydG. The purified HydF partially activated apohydrogenase, suggesting that this maturase is a scaffold protein for H-cluster cofactor assembly and transfer to the hydrogenase [12].

Various recombinant systems have demonstrated active [FeFe] hydrogenase synthesis, both *in vivo* [8], [13]–[15] and *in vitro* [12], [14], [16], and other *in vitro* metalloenzyme systems have shown improved post-translational activation following incubation of the apoproteins with their respective maturases along with exogenous small molecules [17], [18]. Despite these advancements, [FeFe] hydrogenase studies have thus far failed to demonstrate enhanced hydrogenase maturation following small molecule addition, limiting our ability to elucidate the specific biochemistry required for H-cluster cofactor synthesis and installation.

In this work, we describe the first *in vitro* system in which chemical additives stimulate activation of [FeFe] hydrogenase. We recently reported a cell-free system for the production of active hydrogenases [14]. Here, we separate translation and activation into two distinct steps, allowing us to isolate the maturation process and explore it in detail. In agreement with a previous study [16], we noticed that hydrogenase apoprotein was partially activated when added to a crude cell lysate containing the three maturases. However, we observed significantly higher hydrogenase activities when the small molecule components of the cell-free protein synthesis system were also included. This discovery provided a unique opportunity to identify which small molecules play a role in hydrogenase activation and H-cluster biosynthesis.

## Results and Discussion

### The In Vitro System for Enhanced Activation of [FeFe] Hydrogenase

The hydrogenase maturation system contains purified *C. reinhardtii* HydA1 apohydrogenase; dialyzed *E. coli* cell extract containing recombinant HydE, HydF, and HydG hydrogenase maturases from *Shewanella oneidensis* (hereafter referred to as maturase extract); and exogenous small molecules. The eukaryotic *C. reinhardtii* hydrogenase HydA1 was chosen as our model protein given its simplified structure and high degree of *in vivo* solubility. Unlike prokaryotic hydrogenases, algal hydrogenases such as HydA1 have only the C-terminal H-domain and lack N-terminal [4Fe–4S] F-clusters [19]. The *S. oneidensis* maturases HydE, HydF, and HydG were used since previous work established that these proteins are effective in activating HydA1 both *in vivo* [14], [15] and *in vitro* [14].

**Figure 1:**
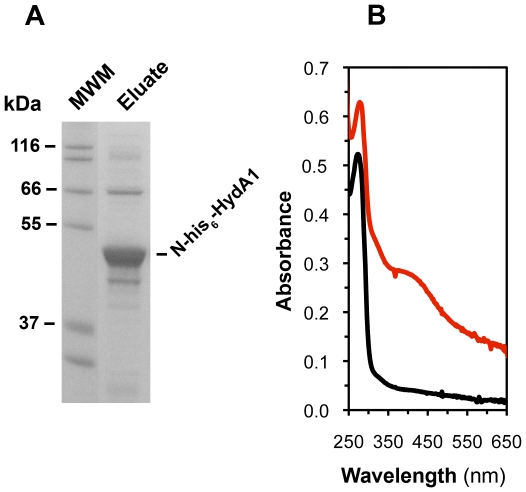
Characterization of purified *C. reinhardtii* HydA1 apohydrogenase. (*Fig. 1A*) SDS-polyacrylamide gel electrophoresis (SDS-PAGE) analysis of pooled elution fractions containing N-his_6_-HydA1 apoprotein (48.4 kDa) following aerobic expression in *E. coli* and subsequent Ni^+2^-affinity chromatography. The molecular weight marker (MWM) is the Mark 12™ protein ladder (Invitrogen). Intermediate lanes of the SDS-polyacrylamide gels were removed, maintaining alignment between the MWM and Eluate lanes. (*Fig. 1B*) UV-visible spectra for 8 µM of as-isolated (black line) and reconstituted (red line) HydA1 apohydrogenase.

HydA1 apohydrogenase was heterologously produced in *E. coli* in the absence of the maturases and purified using immobilized metal-affinity chromatography (IMAC). Pooled fractions contained high purity HydA1 based on SDS-polyacrylamide gels visualized with Coomassie stain (Fig. 1A). Purified apohydrogenase (22.0±4.4 mg HydA1·L^−1^ of culture, n = 3) had 0.3±0.2 mol Fe·mol^−1^ HydA1, which was measured using established methods [20]. For some activation studies, as-isolated apoprotein (apoHydA1) was anaerobically incubated with 1 mM DTT, 0.5 mM Fe(NH_4_)_2_(SO_4_)_2_, and 0.5 mM Na_2_S to reconstitute the [4Fe–4S] cluster. Reconstituted apoprotein (apoHydA1^recon^) preparations are yellow/brown. The UV-visible spectrum for desalted apoHydA1^recon^ (Fig. 1B) shows a broad peak at 400 nm with an *A_400_*:*A_280_* ratio of 0.5, in contrast to the spectrum for apoHydA1. This result indicates the apohydrogenase is properly folded and incorporates the H-domain [4Fe–4S] cluster prior to activation, similar to a previous report [21].

**Figure 2:**
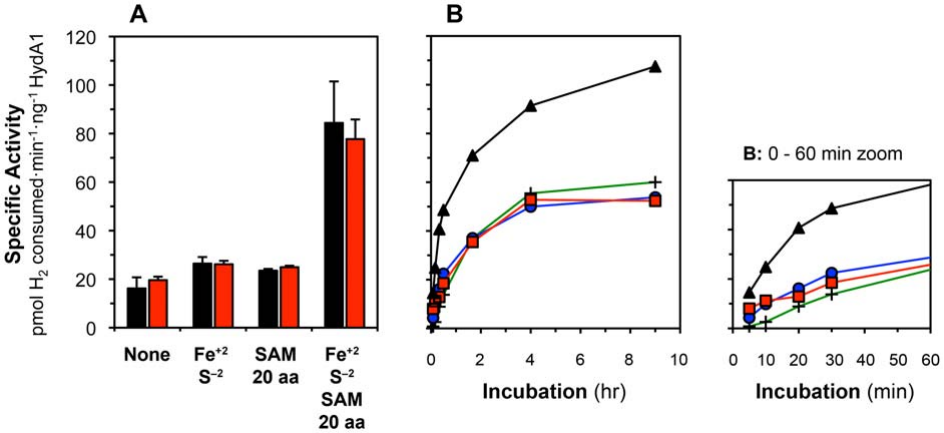
*In vitro* activation of *C. reinhardtii* HydA1 and the effects of exogenous small molecules. 2 µM of HydA1 apoprotein was anaerobically incubated with 50–60% vol·vol^−1^ maturase extract. Exogenous substrates assessed included Fe^+2^ (1 mM), S^−2^ (1 mM), SAM (2 mM), and 20 aa (2 mM of each amino acid). (*Fig. 2A*) When included in reaction mixtures, Fe^+2^ and S^−2^ were added to maturase extracts 2 hr before addition of apoHydA1 (black bars) or apoHydA1^recon^ (red bars). When SAM and 20 aa were included, maturase extracts were incubated with these chemical additives for 1 hr prior to HydA1 addition. Final hydrogenase activities determined after 9 hr of incubation are from n = 2 to 5 independent determinations ± SEM. (*Fig. 2B*) Maturase extracts were reconstituted with Fe^+2^ and S^−2^ for 2 hr (•,▪,▴) or 0 hr (+) before apoHydA1 addition; extracts were also pre-treated with SAM and 20 aa for 1 hr (▴) or 0 hr (▪,•,+) before adding HydA1 apoprotein (as-isolated: •,▴,+; reconstituted: ▪). Data are from n = 2 independent measurements, and standard errors were less than 10% for all data.

Maturase extracts were produced from *E. coli* cells co-expressing HydE, HydF, and HydG in the absence of an [FeFe] hydrogenase. The extracts were dialyzed immediately before use to establish reaction conditions well defined with respect to small molecules. Following anaerobic incubation of apohydrogenase with dialyzed maturase extract, hydrogen uptake activity from activated hydrogenase was determined by measuring methyl viologen reduction rates. Dialyzed maturase extracts were capable of partially activating HydA1 without addition of exogenous molecules (Fig. 2A), as observed with previously described systems [16], [21]. The partial activation may be attributed to [2Fe] sub-clusters produced *in vivo* prior to cell lysis, which are associated with the maturases. No methyl viologen-reducing activity was observed from reaction mixtures when using cell extracts without the maturases or when HydA1 apoprotein was not added.

Ferrous iron (Fe^+2^), inorganic sulfide (S^−2^), SAM, and a mixture of the standard 20 L-amino acids (20 aa) were initially identified as chemical additives contributing to hydrogenase activation. Complementing maturase extracts with 1 mM Fe^+2^, 1 mM S^−2^, 2 mM SAM, and 2 mM of each 20 aa increased hydrogenase activities 4-fold (Fig. 2A). The comparable activities of matured as-isolated and reconstituted apohydrogenase indicate that HydA1 does not require an intact H-domain [4Fe–4S] cluster prior to addition to this system. Exogenous Fe^+2^ and S^−2^ were critical: enhanced HydA1 activation did not occur without both ions despite the presence of SAM and 20 aa. Moreover, Fe^+2^ and S^−2^ were not sufficient to increase hydrogenase activities without SAM and 20 aa. Partial and similar activation of as-isolated and reconstituted apohydrogenase when only SAM and 20 aa were included suggest the Fe^+2^ and S^−2^ are involved in more than just reconstitution of the hydrogenase [4Fe–4S] cluster. Iron and sulfide likely facilitate reconstitution of the maturases' [Fe–S] clusters, which may have been oxidized during aerobic preparation of the cell extracts. Chemical reconstitution of radical SAM proteins using Fe^+2^/Fe^+3^ and S^−2^ has previously been shown to benefit enzyme activity [22], [23]. Additionally, iron and sulfide may be required substrates for *in situ* synthesis of the [2Fe] sub-cluster.

We observed that incubating maturase extracts with Fe^+2^ and S^−2^ before addition of other small molecules and HydA1 apoprotein (termed *extract reconstitution*) provided more consistent data for characterizing the effects of other exogenous substrates. Incubating extracts with SAM and 20 aa following extract reconstitution and before apohydrogenase addition (termed *extract pre-treatment*) led to the immediate onset of maturation as well as maximally enhanced activation (Fig. 2B). When SAM, 20 aa, and HydA1 apoprotein were added to extracts concurrently, hydrogenase maturation was partially compromised, suggesting that such experiments might be useful in exploring the maturation reaction sequence. Extract reconstitution and extract pre-treatment were implemented in all subsequent experiments.

Additional putative small molecule precursors were assessed, although no conditions were identified in which addition of these molecules influenced hydrogenase maturation. While GTPase activity has been attributed to HydF from *Thermotoga maritima* [11], we could not identify any reaction conditions in which exogenous GTP benefited HydA1 activation. Moreover, addition of guanosine diphosphate (GDP) neither enhanced nor inhibited hydrogenase maturation. GTP may play a role in hydrogenase maturation in a biochemical process upstream of those occurring within our system. Carbamoyl phosphate has been identified as a precursor for the cyano ligands associated with the [NiFe] hydrogenase active site [24], [25]. We speculated the H-cluster CN^−^ moieties may also derive from this compound. However, addition of carbamoyl phosphate with exogenous Mg-ATP had no effect on HydA1 activation. Moreover, SDS-PAGE and autoradiography imaging following *in vitro* incubations with [^14^C]-carbamoyl phosphate, Mg-ATP, and maturase extract suggest that carbamoyl phosphate does not covalently associate with HydE, HydF, or HydG. This result could be expected as none of the maturases has a sequence motif characteristic of acyl phosphatases or *O*-carbamoyltransferases like that of the [NiFe] hydrogenase maturase HypF [26], [27]. While thiocyanate reportedly has a strong affinity to an anion-binding cavity of HydE from *T. maritima* [28], conditions were not identified in which thiocyanate or cyanide improved HydA1 maturation. Other *in vitro* studies with [Fe–S] proteins have included reducing agents such as sodium dithionite [17], [18] and dithiothreitol (DTT) [22], [23], [29], though neither compound improved hydrogenase activation in our system. Use of ferrous iron and sulfide ions may obviate the necessity for such reducing agents.

### Exogenous SAM Stimulates In Vitro Hydrogenase Activation

**Figure 3:**
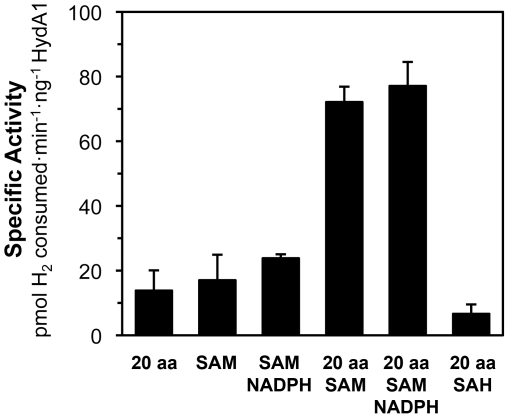
Effects of SAM on *in vitro* HydA1 maturation. Maturase extracts were reconstituted with Fe^+2^ and S^−2^ for 60 min, and then pre-treated for 60 min with the indicated small molecules prior to apoHydA1 addition (3.6–4.6 µM). Reactions mixtures contained 50–70% vol·vol^−1^ maturase extract. Final concentrations of chemical additives were 1 mM Fe^+2^, 1 mM S^−2^, 2 mM of each amino acid (20 aa), 2 mM SAM, 1 mM NADPH, and 2 mM SAH. Hydrogenase activities were measured after 8–9 hr of anaerobic incubation. Data are the average for n = 2 to 4 independent determinations ± SEM.

As shown in Fig. 3, reaction mixtures containing Fe^+2^, S^−2^, 20 aa, and SAM had 5-fold higher HydA1 activities compared to mixtures without SAM. However, neither SAM nor the 20 aa mixture individually enhanced hydrogenase activation (compare Fig. 2B, Fig. 3). The results shown in Fig. 3 suggest that SAM is utilized for *in vitro* HydA1 activation, likely by the maturases HydE and HydG for synthesis of the H-cluster [2Fe] sub-cluster. Several studies have shown that exogenous SAM stimulates *in vitro* biosynthetic reactions catalyzed by radical SAM enzymes. In some cases, a 5′-deoxyadenosyl radical derived from homolytic cleavage of SAM is thought to facilitate abstraction of protons from organic substrates [30], [31]. SAM is also required for the synthesis of NifB-co, a precursor for the nitrogenase FeMo active site cofactor; it has been suggested that radical SAM chemistry also functions to build the FeMo-co [Fe–S] cage [17], [18]. However, no previously reported studies have demonstrated that exogenous SAM improves post-translational [FeFe] hydrogenase activation.

To further explore the stimulatory effect of SAM on hydrogenase activation, the SAM analog *S*-adenosyl homocysteine (SAH) was tested. This analog was not an effective substitute for SAM, likely because SAH contains a less electrophilic sulfur atom.

Reduction of a radical SAM enzyme's [4Fe–4S] cluster to its active state is required for radical chemistry [32]. In some *in vitro* systems, this activation has been shown to further benefit from exogenous NADPH along with SAM addition [29], [31]. Nonetheless, NADPH had no significant effect on final HydA1 activities in our system. Extract reconstitution with Fe^+2^ and S^−2^ may generate reduced [4Fe–4S] clusters associated with the maturases and thus avoid the need for an additional reducing agent.

We have yet to determine how many molecules of SAM are required for activation of one hydrogenase polypeptide or for synthesis of a single H-cluster cofactor. Consumption of multiple SAM molecules per small molecule product for radical SAM-based biochemistry has been reported [33], [34]. Direct detection of the highly reactive 5′-deoxyadenosyl radical has proven difficult, and detection of the allylic analog 5′-deoxyadenosine is generally used to characterize SAM radical chemistry [32]. Our efforts to measure 5′-deoxyadenosine accumulation using reverse-phase HPLC did not show detectable levels in reaction mixtures following HydA1 activation. Future work using purified maturases and higher enzyme concentrations may be more effective for characterizing the radical SAM biochemistry.

### Tyrosine and Cysteine Enhance In Vitro Maturation of HydA1 Hydrogenase

**Figure 4:**
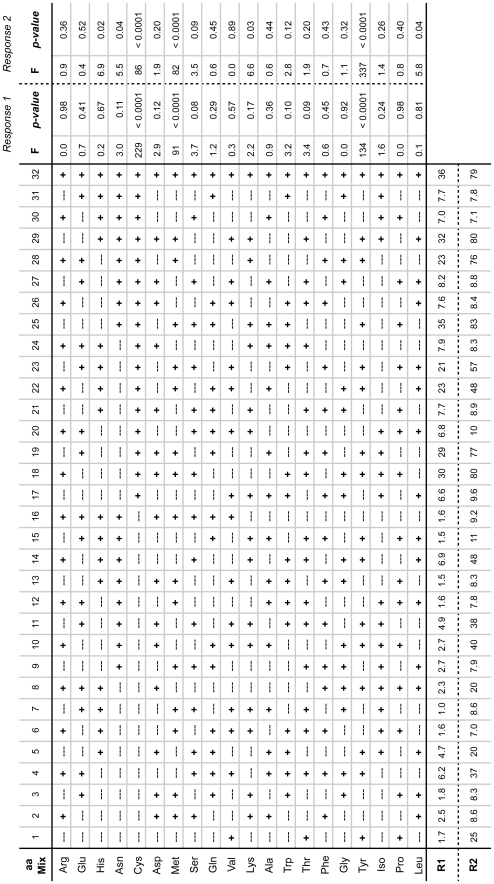
Experimental design for elucidation of amino acids enhancing *in vitro* hydrogenase activation. Amino acid mixtures (aa Mix) were added to hydrogenase maturation reactions to a final concentration of 2 mM for each amino acid. The maturation reaction mixtures contained 60% vol·vol^−1^ maturase extract reconstituted with Fe^+2^ and S^−2^ for 60 min, and then pre-treated with SAM and one of the 32 aa mixtures for 60 min prior to apoHydA1 addition (4.1 µM). HydA1 specific activities were determined at t = 10 min (Response R1) and t = 9 hr (Response R2), and values are expressed as pmol H_2_ consumed·min^−1^·ng^−1^ for n = 1 experiment. ANOVA was performed for both responses to determine F-statistics and *p*-values. The 20 individual amino acid effects were selected for analysis by the regression models. Each F-statistic equals the ratio of mean squares for that particular amino acid (1 degree of freedom) to that of the residuals (11 degrees of freedom). *P*-values represent the statistical significance of the F-statistics.

The requirement for the 20 aa mixture along with Fe^+2^, S^−2^, and SAM (Fig. 3) indicates that one or multiple amino acids may be substrates for hydrogenase activation. A statistical design of experiment approach was adopted to identify amino acids positively or negatively influencing *in vitro* HydA1 activation. Design Expert 7.1 software (Stat-Ease, Inc.) was used to create a 2-level fractional factorial model and for statistical analysis of the data. A 2^20–15^ factorial design with resolution III was selected. 32 combinations of the 20 canonical L-amino acids were constructed using the Design Expert software (Figure 4). Hydrogenase activation reactions contained Fe^+2^, S^−2^, SAM, and one of the 32 amino acid mixtures. Early hydrogenase activities at t = 10 min (Response 1) as well as final hydrogenase activities at t = 9 hr (Response 2) were measured.

Analysis of variance (ANOVA) was used to identify amino acids with statistically significant effects on hydrogenase maturation. All 20 amino acids were included in the regression models. Tyrosine, cysteine, and methionine were identified as having significant positive contributions when analyzing each response, with *p*-values <0.0001 for each amino acid. Cysteine had the most significant effect on early HydA1 activities (Response 1). Tyrosine had the most significant effect on overall HydA1 activities (Response 2), which were 7- to 12-fold higher than the minimum activity. No amino acid had a significant negative effect on HydA1 maturation.

**Figure 5:**
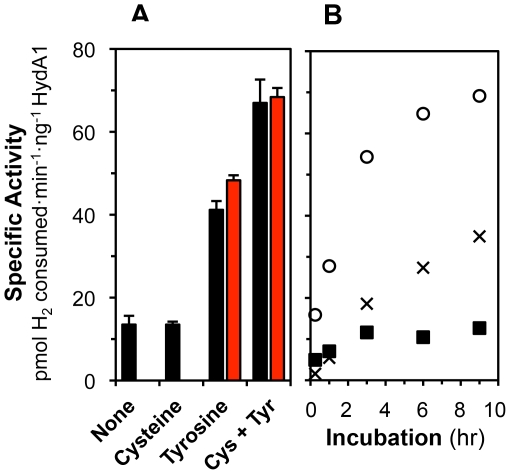
Effects of cysteine and tyrosine on *in vitro* HydA1 activation. Maturase extracts (final concentrations of 50–60% vol·vol^−1^) were reconstituted with Fe^+2^ and S^−2^ for 60 min, and then pre-treated with SAM and amino acids for 60 min prior to addition of apoHydA1 (black bars) or apoHydA1^recon^ (red bars). No additional molecules were added with HydA1 apoprotein (3.6–4.6 µM). Final concentrations of exogenous molecules were as follows: 1 mM Fe^+2^, 1 mM S^−2^, 2 mM SAM, 2 mM cysteine, 2 mM tyrosine, and 2 mM methionine. (*Fig. 5A*) Hydrogenase activities were measured after 8–9 hr of incubation. Data are the average for n = 2 to 5 independent determinations ± SEM. ApoHydA1^recon^ was only tested for mixtures with tyrosine and with cysteine plus tyrosine. Addition of methionine did not enhance hydrogenase activities for all four conditions (data not shown). (*Fig. 5B*) Reaction mixtures included as-isolated apoHydA1, Fe^+2^, S^−2^, SAM, and the following amino acids added as described above: cysteine (▪); tyrosine (×); cysteine and tyrosine (○). Data are the average for n = 2 independent determinations. Standard errors were less than 11% for all data.

With resolution III factorial models, single factor effects are aliased with two-factor interactions. Therefore, our factorial model did not have the ability to assess the independent significance of cysteine, tyrosine, and methionine if two of these molecules have interactive effects on hydrogenase activation. To complete the evaluation, subsequent experiments were done with Fe^+2^, S^−2^, SAM, and the three amino acids. While cysteine and tyrosine individually benefited hydrogenase activation, reaction mixtures with both amino acids had the most effective maturation capability for both maturation kinetics and final activities (Fig. 5A–B). These data suggest that tyrosine and cysteine may have a cooperative interaction for the *in vitro* activation of [FeFe] hydrogenase. However, no conditions were identified in which addition of methionine improved HydA1 activation (data not shown). Examination of the 32 aa mixtures in Figure 4 shows that all 8 mixtures with both tyrosine and cysteine also contained methionine. Thus, the apparent significant effect of methionine indicated by the design of experiment data appears to be a product of the limited discrimination provided by the resolution III factorial model.

We have yet to characterize the biochemical role(s) of cysteine for *in vitro* hydrogenase maturation. We speculate cysteine may be a substrate for synthesis of the H-cluster [2Fe] sub-cluster, specifically as a precursor for the dithiol bridging ligand sulfur atoms. In the absence of cysteine, S^−2^ may substitute for synthesis of the [2Fe] sub-cluster, which could explain why slower and partial HydA1 activation occurred in mixtures containing Fe^+2^, S^−2^, SAM, and tyrosine (Fig. 5B). Alternatively, cysteine could be involved in reconstitution of the maturase [Fe–S] clusters or the hydrogenase [4Fe–4S] cluster; however, apoHydA1^recon^ was matured similarly to apoHydA1 in the absence of exogenous cysteine (Fig. 5A).

### 3,4-Dihydroxy-L-phenylalanine Substitutes for Tyrosine to Stimulate In Vitro HydA1 Activation

**Figure 6:**
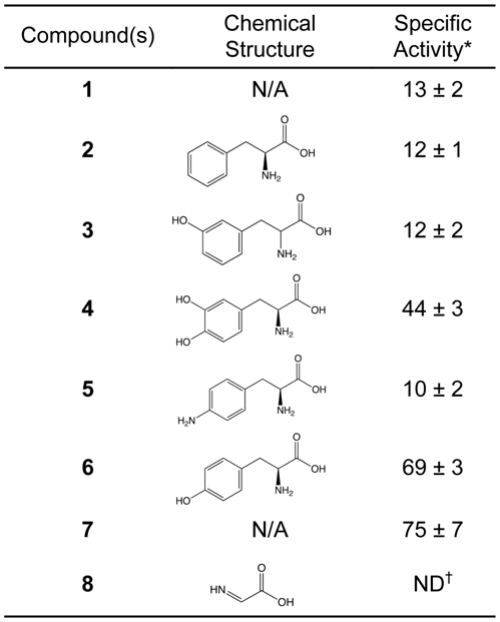
Assessment of tyrosine and tyrosine analogs for *in vitro* hydrogenase activation. Maturase extracts were reconstituted with 1.4 mM Fe^+2^ and 1.4 mM S^−2^ for 60 min and subsequently pre-treated with 2 mM SAM, 2 mM cysteine, and 2 mM of the following amino acid(s) for 60 min prior to apoHydA1 addition (4.6 µM): (1) none, (2) L-phenylalanine, (3) 3-hydroxy-DL-phenylalanine, (4) 3,4-dihydroxy-L-phenylalanine, (5) 4-amino-L-phenylalanine, (6) L-tyrosine (4-hydroxy-L-phenylalanine), (7) 20 aa. Reaction mixtures contained 60% vol·vol^−1^ maturase extract. Hydrogenase activities were measured after 9 hr, and specific activities (*) are expressed as pmol H_2_ consumed·min^−1^·ng^−1^ HydA1. Data are the average for n = 2 independent determinations ± SEM. The chemical structure for dehydroglycine (2-iminoacetic acid) is provided (8). †ND: not determined.

The effects of tyrosine analogs were examined to further investigate the role of tyrosine as a substrate for [FeFe] hydrogenase activation (Figure 6). All reaction mixtures contained Fe^+2^, S^−2^, SAM, and cysteine. Addition of the analog 3,4-dihydroxy-L-phenylalanine partially substituted for tyrosine and improved HydA1 maturation 4-fold. Other tyrosine analogs were ineffective in stimulating hydrogenase activation. Recent *in vitro* work has indicated that thiamine biosynthesis in *E. coli* may require radical SAM chemistry, with tyrosine as a co-substrate [29]. The authors proposed a mechanism by which the 5′-deoxyadenosyl radical generated from SAM abstracts the phenolic hydrogen atom from tyrosine. Subsequent C_α_–C_β_ bond cleavage along with further oxidation of the glycinyl radical results in the formation of dehydroglycine. Thiamine phosphate synthesis using purified enzymes in conjunction with exogenous SAM, tyrosine, and 1-deoxyxylulose-5-phosphate has also been shown [31]. Considering this proposed mechanism, the positive effect of 3,4-dihydroxy-L-phenylalanine on hydrogenase activation could be expected since the molecule has a para-hydroxyl group like that of tyrosine. These results support the suggested role of tyrosine as a substrate for SAM-based radical chemistry to produce intermediates required for synthesis of the H-cluster cofactor [10]. The authors hypothesize that dehydroglycine is a precursor for the [2Fe] sub-cluster dithiomethylamine bridge based on the observation that p-cresol accumulated in HydG-catalyzed reactions between SAM and tyrosine. However, we have yet to distinguish which H-cluster non-protein moieties, if any, are derived from tyrosine. Considering the structure of dehydroglycine (Figure 6) and its carbonyl and imine groups, it is also possible the CO and CN moieties may derive from a dehydroglycine precursor, in addition to the dithiomethylamine bridge as proposed. The observations that Fe^+2^, S^−2^, SAM, cysteine, and tyrosine are sufficient for HydA1 activation further supports this hypothesis as no alternative reaction mechanisms for CO and CN synthesis from these small molecules are apparent.

### Conclusions

In this work, we demonstrate a platform for post-translational activation of an [FeFe] hydrogenase. Utilizing this *in vitro* system, we have shown for the first time the involvement of exogenous small molecules, including ferrous iron, inorganic sulfide, SAM, cysteine, and tyrosine in the activation of an [FeFe] hydrogenase. These results now enable further investigation of H-cluster cofactor biosynthesis using a defined system containing exogenous substrates and purified enzymes.

## Materials and Methods

### Materials and Solution Compositions

Except for isopropyl β-D-1-thiogalactopyranoside (IPTG, Invitrogen), SAM (New England Biolabs), and [^14^C]-carbamoyl phosphate (American Radiolabeled Chemicals, Inc.), all chemicals were obtained from Sigma (Sigma-Aldrich). Commercial aqueous SAM (32 mM stock concentration) contained 10% vol·vol^−1^ ethanol and 5 mM sulfuric acid, pH 2.7. Defined growth medium for fermentations was prepared as previously described [14]. S30 buffer contained 10 mM Tris-acetate, pH 8.0; 14 mM magnesium acetate; and 60 mM potassium acetate. 50 mM sodium phosphate buffer, pH 7.4 with 100 mM NaCl (SP buffer) was used for Ni^+2^-affinity chromatography. 100 mM HEPES/KOH, pH 7.2 with 100 mM NaCl (HP buffer) was used for maturase extract dialysis.

### [FeFe] Hydrogenase and Maturase Expression Vectors

The hydrogenase gene *hydA1* from *C. reinhardtii* as well as the nucleotide sequences *hydGx* and *hydEF* encoding the *S. oneidensis* hydrogenase maturases were PCR amplified from the pK7 *shydA1* and pACYCDuet-1 *hydGxEF* plasmids [14] using Platinum ® Taq DNA Polymerase High Fidelity (Invitrogen). PCR products were digested with restriction enzymes and inserted into expression vectors between a T7 RNA polymerase promoter and terminator using T4 DNA ligase (New England Biolabs). The pY71 vector was used as the parent plasmid for construction of *C. reinhardtii shydA1* expression vectors. The plasmid pY71 *cat* encoding the chloramphenicol acetyl transferase enzyme was synthesized by first PCR amplifying the replication origin from the pUC19 plasmid (Invitrogen), the kanamycin resistance gene from pK7 *cat* [35], and the nucleotide fragment from pK7 *cat* containing the *cat* gene flanked by the T7 RNA polymerase promoter and terminator sequences. These three fragments were ligated using overlapping PCR. The linear PCR product (≈2.5 kb) was digested with BamHI and ligated to form pY71 *cat*. Next, the *shydA1* gene was cloned into the pY71 vector, from which the *cat* gene had been removed. The first 8 codons of *shydA1* were conservatively changed to ATG GCA GCA CCA GCA GCA GAA GCG for reduced secondary structure as predicted using Mfold software to improve *in vitro* translation (shydA1*). pY71 shydA1* was used for addition of an N-terminal 6x-histidine tag, and the *N*-his_6_-shydA1* insert was cloned back into the pY71 vector. Synthesis of the expression vector containing the *S. oneidensis* maturase genes was carried out in two parts. First, the *hydGx* gene segment was cloned into multiple cloning site I of the pACYCDuet-1™ expression vector (Novagen). Next, the *hydEF* gene segment was cloned into multiple cloning site II of pACYCDuet-1–*hydGx*. All expression vectors were confirmed by DNA sequencing and transformed into *E. coli* strain BL21(DE3) (Invitrogen). Transformed cells were selected against kanamycin resistance (40 mg L^−1^) for pK7 and pY71 plasmids, and against chloramphenicol resistance (25 mg L^−1^) for pACYCDuet-1 plasmids.

### Apohydrogenase Expression, Purification, and Characterization

*In vivo* apohydrogenase expression was carried out in the absence of [FeFe] hydrogenase maturases using *E. coli* strain BL21(DE3) pY71 N-his_6_-shydA1*. Cells were initially grown at 30°C in 2 L baffled flasks containing 1 L of LB Miller medium, 40 mg L^−1^ kanamycin, and 250 mg L^−1^ ferric ammonium citrate. Shake flasks were transferred to 20°C shakers at an OD_600_ of 0.2, and L-cysteine was added to 1 mM. After 1 hr (OD_600_≈0.5), IPTG was added to 0.5 mM to induce hydrogenase expression, and cultures were incubated for 12–15 hr at 20°C. Following recombinant hydrogenase expression, cells were pelleted and resuspended in 3 mL of Bug Buster Master Mix lysis solution (Novagen) per gram wet cell mass. Cell suspensions were incubated at 23°C for 30 min and then diluted with 5× SP buffer (10 mM imidazole final concentration). Cell lysates were clarified by centrifugation at 30,000×g and 4°C for 30 min before being loaded onto equilibrated 1 mL HisTrap™ HP Ni^+2^-affinity columns (GE Healthcare). Columns were washed using 5 mL of SP buffer with 40 mM imidazole. Apohydrogenase was eluted using 5 mL of SP buffer with 250 mM imidazole. Eluate fractions containing apoprotein were identified following SDS-PAGE and Coomassie staining. Pooled fractions were dialyzed twice for 3 hr each time against SP buffer with 10% vol·vol^−1^ sucrose. Apohydrogenase aliquots were sealed and stored at −20°C. Protein concentrations were determined with a Qubit fluorometer according to manufacturer's instructions (Invitrogen).

Reconstituted apoprotein solutions were prepared under anaerobic conditions. Solutions were reduced with 1 mM DTT for 15 min, incubated with 0.5 mM Fe(NH_4_)_2_(SO_4_)_2_ for 15 min, and then incubated with 0.5 mM Na_2_S for 2 hr. Reconstituted protein solutions were centrifuged for 15 min at 8,000×g and passed through PD–10 desalting columns (GE Healthcare) equilibrated with HP buffer. Solutions analyzed spectrophotometrically were sealed in quartz cuvettes within the anaerobic chamber. UV-visible spectroscopy was performed using an HP 8425A Diode Array Spectrophotometer (Hewlett Packard). Iron content was measured as previously described [20].

### Production of Maturase Extract for In Vitro Hydrogenase Activation

Recombinant expression of the *S. oneidensis* HydE, HydF, and HydG maturases, and cell-free extract preparation were similar to previously described methods [14]. *E. coli* strain BL21(DE3) pACYCDuet-1–*hydGx*–*hydEF* was cultivated in a 5 L BioFlo 3000 fermentor (New Brunswick Scientific) in 4 L of defined growth medium under oxic conditions at 30°C. The culture pH was maintained at 7.0 using 1 N ammonium hydroxide. Growth medium was supplemented with 25 mg L^−1^ chloramphenicol and 250 mg L^−1^ ferric ammonium citrate. At an OD_600_≈2.0, 1 mM L-cysteine was added, and recombinant maturase expression was induced with 0.5 mM IPTG. After 45 min of induction, the temperature set point was changed to 20°C. When cultures reached 20°C, airflow was switched to 100% nitrogen at 1.5 SLPM to establish strict anoxic conditions. Agitation was reduced from 500 rpm to 75 rpm. Cultures were anaerobically incubated for 12–15 hr at 20°C before cell extract preparation.

All maturase extract preparation steps were carried out under aerobic conditions. Cells were pelleted, resuspended in 1 mL of S30 buffer per gram of wet cell mass, and lysed using a high-pressure EmulsiFlex-C50 homogenizer (Avestin) operated at 15,000–20,000 psi. Cell lysates were clarified by centrifugation at 30,000×g and 4°C for 30 min. Supernatant was collected, frozen with liquid nitrogen, and stored at −80°C until used as maturase extract for *in vitro* hydrogenase activation studies.

### In Vitro Activation of [FeFe] Hydrogenase

Hydrogenase activation reaction mixtures were 25–50 µL in volume and were incubated in 200 µL 8-well PCR strips (E&K Scientific, Inc.). Mixtures contained 50–70% vol·vol^−1^ dialyzed maturase extract, 1–5 µM HydA1 hydrogenase, and exogenous substrates. When included, final concentrations of chemical additives were as follows: 1 mM ferrous ammonium sulfate (Fe^+2^), 1 1 mM sodium sulfide (S^−2^), 2 mM SAM, a mixture 20 standard L-amino acids at 2 mM each, 2 mM SAH, 1 mM NADPH, 2–20 mM magnesium chloride, 1–10 mM ATP, 2 mM cysteine, 2 mM tyrosine, 2 mM methionine, 2 mM phenylalanine, 2 mM 4-amino-L-phenylalanine, 2 mM 3-hydroxy-DL-phenylalanine, 2 mM 3,4-dihydroxy-L-phenylalanine, 2 mM GTP, 2 mM GDP, 1–5 mM carbamoyl phosphate, 2 mM sodium thiocyanate, 2 mM sodium cyanide, 1 mM DTT, and 1 mM sodium dithionite.

Generally, hydrogenase maturation reactions consisted of four phases, with all procedures carried out in an anaerobic chamber (Coy Laboratory Products) containing 98% N_2_ and 2% H_2_. *Phase 1, dialysis*: 0.5–2.0 mL of maturase extract was buffer exchanged three times (3 hr, 3 hr, overnight) against 0.75 L of HP buffer at 6°C using 6–8 kD MWCO RC dialysis tubing (Spectrum Laboratories, Inc.). Dialyzed maturase extracts were used immediately to avoid variability from freezing and thawing. *Phase 2, extract reconstitution*: Fe^+2^ and S^−2^ were incubated with dialyzed extracts for 60 min at 26°C before pre-treatment with small molecules. *Phase 3, extract pre-treatment*: reconstituted extracts were incubated with defined sets of exogenous substrates for 60 min at 26°C before apohydrogenase addition. *Phase 4, hydrogenase activation*: either as-isolated or reconstituted apohydrogenase was added to pre-treated maturase extracts, and reaction mixtures were incubated at 26°C until assayed for hydrogenase activity. Hydrogenase activity was determined with a H_2_ consumption and methyl viologen reduction assay as previously described [14], with the modification that spectrophotometric measurements were performed at 26°C instead of 37°C.
